# Preoperative Platelet-to-Lymphocyte Ratio (PLR) for Predicting the Survival of Stage I-III Gastric Cancer Patients with a MGC Component

**DOI:** 10.1155/2021/9678363

**Published:** 2021-05-03

**Authors:** Ziyu Zhu, Jialiang Gao, Zhixin Liu, Chunfeng Li, Yingwei Xue

**Affiliations:** ^1^Department of Gastrointestinal Surgery, Harbin Medical University Cancer Hospital, 150 Haping Road, Nangang District, Harbin 150081, China; ^2^Department of Gynecology, Harbin Medical University Cancer Hospital, 150 Haping Road, Nangang District, Harbin 150081, China

## Abstract

**Background:**

The preoperative platelet-to-lymphocyte ratio (PLR) evaluates the prognosis of gastric cancer patients. However, whether preoperative PLR may be used to evaluate the prognosis of mucinous gastric carcinoma (MGC) patients is poorly investigated. The present study evaluated the effect of preoperative PLR on overall survival in gastric cancer patients with a mucinous component.

**Methods:**

A total of 336 MGC were enrolled in this study, and the characteristics of the tumor, including pathological features and clinical data, were retrospectively analyzed.

**Results:**

A high PLR was associated with larger tumor size, advanced tumor invasion, lymph node metastasis, advanced TNM stage, tumor location, total gastrectomy, low hemoglobin level, low albumin level, high fibrinogen level, high platelet level, and high neutrophil-to-lymphocyte ratio (NLR, all *P*′s < 0.05). Multivariate analysis identified age (HR = 1.876; 95% CI 1.361-2.585, *P* < 0.001), TNM stage (HR = 2.350; 95% CI 1.216-4.542, *P* = 0.011), globulin (HR = 1.520; 95% CI 1.067-2.165, *P* = 0.020), total gastrectomy (HR = 0.537; 95% CI 0.373-0.772, *P* = 0.001), and PLR (HR = 1.582; 95% CI 1.066-2.348, *P* = 0.023) as independent prognostic factors for OS.

**Conclusion:**

Preoperative PLR is related to pathological features and may independently evaluate the survival of MGC. Therefore, preoperative PLR may help physicians develop treatment plans and evaluate survival in these patients.

## 1. Introduction

Gastric cancer is the 5th most common cancer worldwide and the 3rd deadliest malignancy. Approximately 783,000 people died of gastric carcinoma in 2018 [1]. Although the research on gastric cancer is progressing, the histological classification of gastric cancer remains ambiguous and controversial [2].

Mucinous gastric carcinoma (MGC) is a special histological type of gastric cancer, which is defined using different standards, such as diffuse-type by Lauren, infiltrative-type by Ming, and high-grade by the WHO. Mucinous carcinoma accounts for 2–6% of all gastric cancers [3–5]. The WHO criteria for gastric cancer are a relatively large amount of extracellular mucin (>50% of tumor volume) within tumors, and it is defined as pMGC. A relatively small amount of extracellular mucin (10%-50% of tumor volume) within tumors is defined as mMGC [6]. There are few studies on pMGC. Some of these studies reported poor prognosis, and other studies showed no difference in prognosis between pMGC and other histological types of gastric cancer. Therefore, the clinicopathological features and prognosis of pMGC are controversial. Mucinous gastric carcinoma is rarely reported. Therefore, the study of gastric cancer (pMGC and mMGC) containing extracellular mucin in tumor tissues has important clinical significance [7–9].

Tumor-associated inflammatory cells and inflammatory mediators received increasing attention in recent years [10]. Peripheral platelets, leukocytes, lymphocytes, and neutrophils promote inflammatory responses [11]. Increasing studies evidenced that the systemic inflammatory response was related to the prognosis of a variety of cancer patients [12, 13]. The inflammatory response indicators include PNI (prognosis nutrition index), NLR (neutrophil-to-lymphocyte ratio), and PLR (platelet-to-lymphocyte ratio). PLR is a reliable factor in the prediction of the cancer prognosis, and it is easily obtained [14]. Cumulative evidence suggested that PLR was an independent risk factor for gastric cancer prognosis and survival. However, there is little data on the prognostic significance of gastric cancer with extracellular mucin in the pathological tissues [15, 16].

The present study evaluated the effect of preoperative PLR on overall survival in gastric cancer patients with extracellular mucin in the pathological tissues.

## 2. Methods

### 2.1. Patients

A total of 336 patients who were diagnosed with gastric cancer with a mucinous component and underwent gastrectomy between August 2001 and December 2013 at the Harbin Medical University Cancer Hospital in Heilongjiang, China, were included. The following inclusion criteria were used: (1) all patients diagnosed with gastric cancer with a mucinous component via pathological examination of postoperative tissue specimens, (2) receipt of radical surgery (R0/R1/R2 resection), (3) absence of preoperative neoadjuvant radiotherapy or chemotherapy, (4) patients not taking nutrition replacement therapy or any drugs that may affect the serum markers before blood sample collection, and (5) complete clinicopathological and follow-up data.

### 2.2. Clinical Data Collection and Processing

The laboratory examination samples were collected within 1 week before surgery, which included leukocytes (10^9^/L), lymphocytes (10^9^/L), platelets (10^9^/L), neutrophils (10^9^/L), serum fibrinogen (g/L), serum albumin (g/L), serum globulin (g/L), and hemoglobin (g/L). Other clinical pathological factors included age, sex, pathologic type [6], tumor size, tumor location, R0/(R1/R2) resection, chemotherapy after surgery, tumor infiltration, lymph node metastasis, and TNM stage [17].

The present study calculated PLR and NLR as follows: PLR = P/L and NLR = N/L, where L, P, and N indicate lymphocytes, platelets, and neutrophils, respectively.

### 2.3. Follow-Up

Patients were followed up via telephone or outpatient review. The follow-up was every 3 months in the first two years and every 6 months thereafter. The overall survival (OS) time was from the date of surgery to the death date or last date of follow-up. The follow-up was from December 2001 to January 2018, and the median follow-up time of the patients was 37 (3-60) months. All patients discontinued the follow-up after 5 years of follow-up.

### 2.4. Statistical Analysis

SPSS 21.0 software (IBM, Chicago, IL, USA) was used for all data analyses. The ROC curve was used to determine the optimal cut-off values for prognostic indicators. Categorical variables were compared using the chi-squared test. Survival analysis was analyzed using the Kaplan-Meier method, and the log-rank test was used to compare the survival difference. Multivariate prognosis analysis was performed using a Cox proportional hazards regression model. *P* < 0.05 was considered statistically significant.

## 3. Results

### 3.1. Patient Characteristics

The present study included 336 patients; 237 (70.5%) were men, and 99 (29.5%) were women. The median age was 59 years (range 32-86 years). The median follow-up duration was 37 months (range 3–60 months). At the last follow-up, 185 (55.1%) patients had died, and 151 (44.9%) patients were still alive.

### 3.2. The Optimal Cut-Off Value for Prognostic Factor

The ROC curves based on 5-year OS rates were used to define the cut-off for tumor size (0.675, *P* < 0.001), leukocytes (0.519, *P* = 0.557), neutrophils (0.552, *P* = 0.103), hemoglobin (0.573, *P* = 0.021), fibrinogen (0.595, *P* = 0.003), albumin (0.587, *P* = 0.006), globulin (0.522, *P* = 0.480), platelets (0.567, *P* = 0.036), NLR (0.583, *P* = 0.009), and PLR (0.609, *P* = 0.001), and the areas under the ROC curve (AUC) were disposed above. The optimal cut-offs were determined to be 5.25 for tumor size, 6.17 for leukocytes, 3.31 for neutrophils, 125.1 for hemoglobin, 3.31 for fibrinogen, 40.1 for albumin, 29.2 for globulin, 243 for platelets, 1.6 for NLR, and 133 for PLR. Consequently, patients were divided into two groups based on the optimal cut-off value. The low group was less than the optimal cut-off value, and the high group was equal to or higher than the optimal cut-off value (Table 1).

### 3.3. Relationship between PLR and Clinicopathological Characteristics

The high PLR was associated with tumor diameter, advanced tumor invasion, lymph node metastasis, advanced TNM stage, tumor location, total gastrectomy, low hemoglobin level, low albumin level, high fibrinogen level, high platelet level, and high NLR (all *P*′s < 0.05, Table 2).

### 3.4. Univariate and Multivariate Survival Analyses

Univariate analysis identified age ≥ 59 years (*P* = 0.001), R1/R2 resection (*P* < 0.001), tumor size ≥ 5.25 cm (*P* < 0.001), advanced tumor invasion (*P* < 0.001), lymph node metastasis (*P* < 0.001), advanced TNM stage (*P* < 0.001), tumor location (*P* < 0.001), neutrophils ≥ 3.3 (*P* = 0.030), hemoglobin < 125.1 (*P* = 0.009), fibrinogen ≥ 3.31 (*P* = 0.005), albumin < 40.1 (*P* < 0.001), globulin ≥ 29.2 (*P* = 0.039), platelets ≥ 243 (*P* = 0.010), total gastrectomy (*P* < 0.001), NLR ≥ 1.6 (*P* = 0.002), and PLR ≥ 133 (*P* < 0.001) as prognostic factors for poor OS (Table 3).

**Figure 1:**
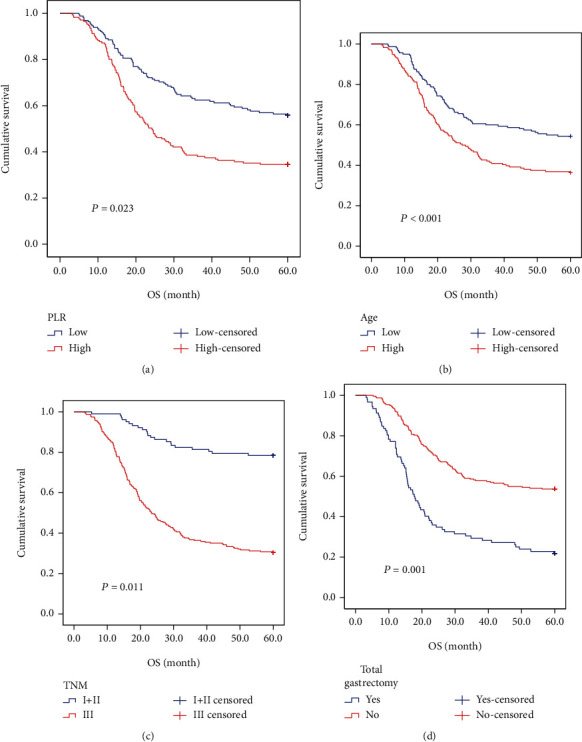
The Kaplan-Meier survival curve for gastric cancer patients with MGC component: (a) PLR: <133 or ≥133; (b) age: <59 years or ≥59 years; (c) pTNM stage: I+II stage and III stage; (d) total gastrectomy: yes or no.

Multivariate analysis identified age (HR = 1.876; 95% CI 1.361-2.585, *P* < 0.001), TNM stage (HR = 2.350; 95% CI 1.216-4.542, *P* = 0.011), globulin (HR = 1.520; 95% CI 1.067-2.165, *P* = 0.020), total gastrectomy (HR = 0.537; 95% CI 0.373-0.772, *P* = 0.001), and PLR (HR = 1.582; 95% CI 1.066-2.348, *P* = 0.023) were independent prognostic factors for OS (Table 3, Figure 1).

### 3.5. Subgroup Analysis

**Figure 2:**
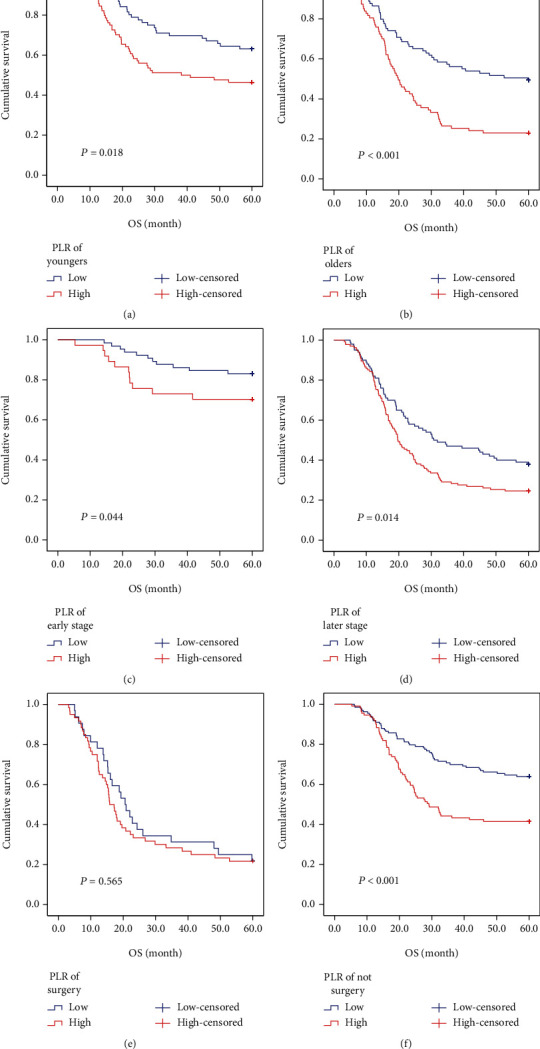
Kaplan-Meier analysis of OS for patients with gastric cancer with opposite PLR: (a) of younger age (<59 years) group; (b) of older age (≥59 years) group; (c) at TNM stage I+II; (d) at TNM stage III; (e) in total gastrectomy resection group; (f) in nontotal gastrectomy resection group.

We further investigated the prognostic value of PLR relative to different TNM stages, mucinous component, age, and use of total gastrectomy; a forceful association between PLR and OS was found irrespective of mucinous component (*P* < 0.001 for <50%; *P* = 0.027 for ≥50%) and age (*P* < 0.001 for <59; *P* = 0.018 for ≥59). PLR was valuable in the prognosis of patients with stage III (*P* = 0.014) and nontotal gastrectomy (*P* < 0.001). For patients with stage I+II (*P* = 0.099) and total gastrectomy (*P* = 0.565), no significant association of PLR with OS was identified. The prognosis for patients with stage I+II in the low PLR group (5-year OS rates was 83%) had a better trend than the high PLR group (5-year OS rates was 70.3%), as illustrated in Figure 2.

## 4. Discussion

PLR is a widely used, repeatable, and inexpensive laboratory hematological marker, and it has become a hot topic in tumor research. Cumulative studies showed that the value of PLR was related to the early diagnosis, recurrence prediction, and prognosis evaluation of tumors, including gastric cancer, colorectal cancer, and breast cancer [14, 18, 19]. However, whether it may become a new reliable indicator for the early detection and prediction of overall survival of mucinous gastric carcinoma (i.e., the pathological tissue contains extracellular mucin in gastric cancer) remains to be studied.

PLR was an independent risk factor that affected the prognosis of mucinous gastric carcinoma in our study. It effectively predicted the overall survival of patients, and it was closely related to the clinical and pathological characteristics. Preoperative blood samples revealed that high PLR was more common in mucinous gastric carcinoma with lower hemoglobin and serum albumin.

Several studies showed that patients with advanced gastric cancer were more prone to anemia and reduced nutritional status than patients with early gastric cancer, and low levels of hemoglobin and serum albumin also indicated a poor prognosis for patients with gastric cancer [20, 21]. Among the prognostic factors after surgery, we found that mucinous gastric carcinoma with high PLR had larger tumor diameter and greater probability of total gastrectomy and was more prone to serosal infiltration and lymph node metastasis. Serosal infiltration and lymph node metastasis are important components of the TNM stage. Judgment and comprehensive analysis of patient data found that stage III PLR of mucinous gastric carcinoma was higher than stage I+II [22]. Larger tumor diameter is also a highly malignant indicator of gastric cancer, and the larger tumor diameters tend to have higher PLR [23, 24]. Similar findings are demonstrated in other cancers [25, 26]. Lan et al. tested PLR levels in the blood of patients with lung cancer and found that the values of PLR were related to the pathological characteristics [27]. Therefore, we deemed that PLR may be a valid indicator to reflect the malignant degree of mucinous gastric carcinoma.

The research value of PLR in gastric cancer has received more attention from scholars in recent years. A meta-analysis of 13 studies with a total of 6280 patients indicated that high PLR was an essential prognostic blood marker for poor overall survival in patients with gastric cancer [22]. Some studies have shown that PLR is of great value in the prognosis assessment of early gastric cancer, advanced gastric cancer, surgical treatment of gastric cancer, chemotherapy for gastric cancer, and neoadjuvant chemotherapy for gastric cancer [15, 28, 29]. Our study supports the value of PLR in the prognosis assessment of mucinous gastric carcinoma. Systemic inflammatory responses contribute to the initiation, development, and metastasis of tumors and affect the prognosis of patients in multiple aspects [30]. The mechanism of PLR in tumors may first involve the presence of thrombocytosis, which tends to represent a cancer-related inflammation response [31]. Platelets contribute to tumor progression, including angiogenesis, tumor growth, and metastasis, via VEGF, PAF, and PDGF [32]. Second, PF4/CXCL4 (platelet factor 4) regulates tumor angiogenesis and inflammation within the tumor environment, and the other platelet-associated chemokines also have the same function, such as CTAP-III (connective tissue-activating peptide III) [32]. Lymphocytes play a significant role in the antitumor immune responses and cancer immunosurveillance process. Lymphocytes prevented the development of spontaneous epithelial carcinomas and chemically induced sarcomas via cooperation with IFN-*γ* [33, 34]. To a certain extent, PLR accurately reflected the trend of changes in lymphocytes and platelets in the blood of tumor patients, which indicates that it may have an important role in evaluating the prognosis of mucinous gastric carcinoma.

We found that the 5-year survival rate of the low-PLR group was significantly higher in the MGC patients than the high-PLR group (55.6% vs. 34.5%), and the PLR was associated with OS-related univariate and multivariate analyses and had statistical significance. Therefore, PLR is an independent prognostic factor that affects the prognosis of patients with mMGC and pMGC. Deng et al. showed that high PLR predicted that non-small-cell lung cancer patients who underwent surgery tended to have a poor prognosis [35]. Toda et al. also confirmed that elevated PLR was an independent prognostic factor for OS of patients with liver cancer [36]. Our conclusions are also supported by these studies. Despite the strong predictive power of pTNM staging, their access requires surgery. However, PLR may be calculated only from routine blood examination, which is convenient, simple, economical, and quick. We compared the prognostic value of several common indicators in blood samples using the ROC curve and found that the AUC region of the PLR was larger, which means that the PLR had a satisfactory prognostic value. The subgroup analysis revealed that PLR had a significant association with OS in pMGC or mMGC and age (no less than 59 years). The prognosis survival between the high and low PLR of stage III patients was statistically significant, but not in stage I+II, and the prognosis of the patients with low-grade PLR (average survival time of 54 months) was better than patients with high-grade PLR (mean survival 48 months). These results strongly support our conclusion that PLR is an independent factor that affects the prognosis of patients with mucinous gastric carcinoma.

Blood indicators are important for early diagnosis, individual treatment, and prognosis assessment. Due to its reproducibility, easy access, and low cost, an increasing number of scholars are paying more attention to gastric cancer blood markers in recent years. However, their research was aimed at the whole body of gastric cancer, and our research focused on special pathological histology of gastric cancer with a mucinous component. Therefore, our research has important clinical value. PLR may be used as one of the reliable reference indicators for prognosis evaluation of mucinous gastric carcinoma in clinical applications. The optimal interception value of PLR is controversial, and the mechanism of PLR rise and lymphocyte reduction in preoperative tumor patients remains unclear. This limitation was also a flaw in our research.

The limitations of this study are the small sample size and lack of DFS (disease-free survival). However, because of the complete and credible data on OS, the value of PLR for the prognosis of patients with MGC is sufficient and convincing. The subgroup analysis combining different clinical characteristics corroborates the research results. Similar studies have consistent problems with the use of peripheral blood cell counts and derived indices. These cells change rapidly in the human body due to their susceptibility to a variety of physiological and environmental factors. Although we used the relatively effective ROC method to determine the best cut-off value, other researchers tend to use the mean, median, or reference range for measurement. Therefore, our prediction model may be biased. Further research will probe the mechanism of how the higher PLR promoted MGC using immunohistochemistry or animal experiments and accumulate more clinical data of MGC patients to verify the association between PLR and MGC.

In summary, mucinous adenocarcinoma should be a special type of gastric cancer, and changes in PLR should be noted in clinical work because it may contribute to planning in review time and the evaluation prognosis of mucinous gastric carcinoma.

## 5. Conclusion

The high PLR in gastric cancer patients with mucinous component was connected with larger tumor size, advanced tumor invasion, lymph node metastasis, and advanced TNM stage in our study, but it was also associated with a low hemoglobin level, low albumin level, and high NLR. More importantly, we found that PLR was an independent prognostic factor that affected the prognosis of patients with mMGC and pMGC. Our results suggest that physicians may use PLR as a biomarker in the evaluation of the prognosis of gastric cancer patients with a mucinous component.

## Figures and Tables

**Table 1:** The optimal thresholds for prognostic factors by the ROC curves.

Variables	Threshold	Sensitivity	Specificity	AUC area (95% CI)	*P* value
Tumor size	5.25	0.667	0.603	0.675 (0.617-0.732)	<0.001^∗^
Leukocyte	6.17	0.489	0.603	0.519 (0.457-0.580)	0.557
Neutrophil	3.31	0.591	0.517	0.552 (0.490-0.613)	0.103
Hemoglobin	125.1	0.636	0.511	0.573 (0.512-0.634)	0.021
Fibrinogen	3.31	0.554	0.603	0.595 (0.535-0.656)	0.003^∗^
Albumin	40.1	0.649	0.532	0.587 (0.526-0.648)	0.006^∗^
Globulin	29.2	0.285	0.808	0.522 (0.461-0.584)	0.480
Platelet	243	0.554	0.583	0.567 (0.505-0.628)	0.036^∗^
NLR	1.6	0.731	0.417	0.583 (0.522-0.044)	0.009^∗^
PLR	133	0.608	0.609	0.609 (0.549-0.670)	0.001^∗^

^∗^ *P* < 0.05; NLR = neutrophil-to-lymphocyte ratio; PLR = platelet-to-lymphocyte ratio.

**Table 2:** The clinicopathological characteristics of 336 patients with gastric cancer.

Variables	PLR < 133 (165 cases)	PLR ≥ 133 (171 cases)	X2 value	*P* value
*Sex*			1.221	0.296
Male	121	116		
Female	44	55		
*Age (years)*			0.316	0.574
<59	76	84		
≥59	89	87		
*mMGC/pMGC*			0.889	0.346
mMGC	116	112		
pMGC	49	59		
*Tumor size (cm)*			12.861	<0.001^∗^
<5.25	91	61		
≥5.25	74	110		
*Chemotherapy*			2.427	0.119
Yes	69	86		
No	96	85		
*R0/R1&2 resection*			2.594	0.107
R0	143	137		
R1/R2	22	34		
*pT*			15.887	0.001^∗^
T1	15	3		
T2	25	12		
T3	19	24		
T4	106	132		
*pN*			15.730	0.003^∗^
N0	45	27		
N1	35	28		
N2	39	38		
N3a	33	45		
N3b	13	33		
*pTNM*			12.523	<0.001^∗^
I+II	65	37		
III	100	134		
*Tumor location*			12.906	0.024^∗^
L	109	86		
M	16	31		
U	18	17		
LM	13	25		
MU	6	5		
LMU	3	7		
*Leukocyte*			3.768	0.052
<6.17	82	103		
≥6.17	83	68		
*Neutrophil*			2.609	0.106
<3.31	83	71		
≥3.31	82	100		
*Hemoglobin*			35.619	<0.001^∗^
<125.1	46	103		
≥125.1	119	68		
*Fibrinogen*			10.791	0.001^∗^
<3.31	100	73		
≥3.31	65	98		
*Albumin*			4.031	0.045^∗^
<40.1	65	86		
≥40.1	100	85		
*Globulin*			2.121	0.145
<29.2	119	135		
≥29.2	46	36		
*Platelet*			60.100	<0.001^∗^
<243	119	51		
≥243	46	120		
*Total gastrectomy*			10.402	0.001^∗^
Yes	32	60		
No	133	111		
*NLR*			56.385	<0.001^∗^
<1.6	88	25		
≥1.6	77	146		

^∗^ *P* < 0.05; L = lower; M = middle; U = upper; NLR = neutrophil-to-lymphocyte ratio; MGC = mucinous gastric carcinoma.

**Table 3:** The analysis of prognosis factors in 336 gastric cancer patients with MGC component by univariate and multivariate.

Variables		Univariate analysis		Multivariate analysis	
		5-YSR (%)	*P* value	HR (95% CI)	*P* value
Sex	Male	42.2	0.161	—	—
	Female	51.5			
Age (years)	<59	54.4	0.001	1.876 (1.361-2.585)	<0.001^∗^
	≥59	36.4			
R0/R1&2 resection	R0	51.1	<0.001	1.384 (0.927-2.067)	0.112
	R1/R2	14.3			
Tumor size (cm)	<5.25	59.9	<0.001	0.884 (0.608-1.286)	0.520
	≥5.25	32.6			
pMGC/mMGC	mMGC	47.4	0.211	—	—
	pMGC	39.8			
Chemotherapy	Yes	47.1	0.191	—	—
	No	43.1			
T-stage	T1	88.9	<0.001	1.289 (0.948-1.753)	0.106
	T2	78.4			
	T3	48.8			
	T4	35.7			
N-stage	N0	80.6	<0.001	1.136 (0.962-1.341)	0.132
	N1	49.2			
	N2	40.3			
	N3a	29.5			
	N3b	17.4			
TNM	I+II	78.4	<0.001	2.350 (1.216-4.542)	0.011^∗^
	III	30.3			
Tumor location	L	54.4	<0.001	1.103 (0.982-1.238)	0.097
	M	38.3			
	U	28.6			
	LM	34.2			
	MU	18.2			
	LMU	20.0			
Leukocyte	<6.17	49.2	0.077	—	—
	≥6.17	39.7			
Neutrophil	<3.31	50.6	0.030	1.174 (0.818-1.684)	0.384
	≥3.31	40.1			
Hemoglobin	<125.1	36.9	0.009	1.096 (0.785-1.528)	0.591
	≥125.1	51.3			
Fibrinogen	<3.31	52.6	0.005	0.897 (0.655-1.230)	0.500
	≥3.31	36.8			
Albumin	<40.1	35.1	<0.001	0.761 (0.553-1.046)	0.092^∗^
	≥40.1	53.0			
Globulin	<29.2	48.0	0.039	1.520 (1.067-2.165)	0.020^∗^
	≥29.2	35.4			
NLR	<1.6	55.8	0.002	1.012 (0.662-1.548)	0.956
	≥1.6	39.5			
Platelet	<243	51.8	0.010	0.895 (0.619-1.293)	0.554
	≥243	38.0			
PLR	<133	55.6	<0.001	1.582 (1.066-2.348)	0.023^∗^
	≥133	34.5			
Total gastrectomy	Yes	21.7	<0.001	0.537 (0.373-0.772)	0.001^∗^
	No	53.7			

^∗^ *P* < 0.05; 5-YSR = 5-year survival rate; L = lower; M = middle; U = upper; NLR = neutrophil-to-lymphocyte ratio; PLR = platelet-to-lymphocyte ratio; MGC = mucinous gastric carcinoma.

## Data Availability

The patient clinical data used to support the findings of this study have not been made available because the patients' privacy needs to be kept confidential.
